# Exploring life-space in the nursing home. An observational longitudinal study

**DOI:** 10.1186/s12877-021-02345-0

**Published:** 2021-06-29

**Authors:** Karen Sverdrup, Sverre Bergh, Geir Selbæk, Jūratė Šaltytė Benth, Bettina Husebø, Irene Mari Røen, Pernille Thingstad, Gro Gujord Tangen

**Affiliations:** 1 Norwegian National Advisory Unit on Ageing and Health, Vestfold Hospital Trust, Aldring og Helse, Postboks 2136, 3103, Tønsberg, Norway; 2 Department of Geriatric Medicine, Oslo University Hospital, Oslo, Norway; 3 Department of Interdisciplinary Health Sciences, Institute of Health and Society, Faculty of Medicine, University of Oslo, Oslo, Norway; 4 Research Centre for Age-Related Functional Decline and Disease, Innlandet Hospital Trust, Brumunddal, Norway; 5 Institute of Clinical Medicine, Faculty of Medicine, University of Oslo, Oslo, Norway; 6 Institute of Clinical Medicine, Campus Ahus, University of Oslo, Oslo, Norway; 7 Health Services Research Unit, Akershus University Hospital, Lørenskog, Norway; 8 Department of Global Public Health and Primary Care, Centre for Elderly and Nursing Home Medicine, University of Bergen, Bergen, Norway; 9 Department of Nursing Home Medicine, Bergen, Norway; 10 Centre for Development of Institutional and Home Care Services, Innlandet (Hedmark), Norway; 11 Department of Neuromedicine and Movement Science, Faculty of Medicine and Health Sciences, NTNU, Trondheim, Norway

**Keywords:** Life-space, Long-term care facility, Dementia, Physical performance

## Abstract

**Background:**

Traditional performance-based measurements of mobility fail to recognize the interaction between the individual and their environment. Life-space (LS) forms a central element in the broader context of mobility and has received growing attention in gerontology. Still, knowledge on LS in the nursing home (NH) remains sparse. The aim of this study was to identify LS trajectories in people with dementia from time of NH admission, and explore characteristics associated with LS over time.

**Methods:**

In total, 583 people with dementia were included at NH admission and assessed biannually for 3 years. LS was assessed using the Nursing Home Life-Space Diameter. Association with individual (age, sex, general medical health, number of medications, pain, physical performance, dementia severity, and neuropsychiatric symptoms) and environmental (staff-to-resident ratio, unit size, and quality of the physical environment) characterises was assessed. We used a growth mixture model to identify LS trajectories and linear mixed model was used to explore characteristics associated with LS over time.

**Results:**

We identified four groups of residents with distinct LS trajectories, labelled Group 1 (*n* = 19, 3.5%), Group 2 (*n* = 390, 72.1%), Group 3 (*n* = 56, 10.4%), Group 4 (*n* = 76, 14.0%). Being younger, having good compared to poor general medical health, less severe dementia, more agitation, less apathy, better physical performance and living in a smaller unit were associated with a wider LS throughout the study period.

**Conclusion:**

From NH admission most NH residents’ LS trajectory remained stable (Group 2), and their daily lives unfolded within their unit. Better physical performance and less apathy emerged as potentially modifiable characteristics associated with wider LS over time. Future studies are encouraged to determine whether LS trajectories in NH residents are modifiable, and we suggest that future research further explore the impact of environmental characteristics.

**Supplementary Information:**

The online version contains supplementary material available at 10.1186/s12877-021-02345-0.

## Background

Limitations in mobility is associated with an abundance of adverse health outcomes and identifying and preventing mobility impairments is a global public-health priority [1]. Traditionally, performance-based measurements, such as gait speed, balance, and muscle strength have been used to assess mobility in older adults. However, these fail to recognize the interaction between the individual and their environment [2, 3]. Life-space (LS) forms a central element in a broader context of mobility [2] and captures the extent and frequency of movement across life zones, from the bedroom outward, during a set time period [4–7]. LS is related to a large set of factors in the environment including social support, social network and participation [3, 8–10]. Various questionnaires have been developed for assessing LS [4–7], and LS has received growing attention in gerontology the last decade [8].

Several studies have examined LS and its associated characteristics in community-dwelling older adults. These indicate that restricted LS is associated with older age, more severe depression, apathy, comorbidity, cognitive impairment, impairments in physical performance, and environmental barriers [7, 11–15]. Further, restricted LS can predict healthcare utilization [16], nursing-home (NH) admission [17], falls [18], and mortality [19, 20]. Yet, LS has received scant attention in NH care environments.

Influenced by the increased proportion of older adults in society and growing numbers of dependent older adults, the demand for NH care will remain high [21]. NH residents are a highly vulnerable population with multiple risk factors for restricted LS. The prevalence of dementia among this population is high (58–84%) [22, 23], and most residents experience impairments in physical performance [24] and neuropsychiatric symptoms (NPS) [23]. Mobility within NHs has several unique features [5, 25]. Environmental characteristics such as physical environment (e.g., lighting, maintenance, cleanliness); architectural features (e.g., layout and size of rooms, and common spaces); and schedules and care routines (e.g., staff availability, mealtimes) may influence LS [26]. The role of environmental characteristics in NHs has emerged as an important care component associated with better quality of life, improved activities of daily life, and reduction in NPS [27].

The evidence of LS in NH residents is sparse, but cross-sectional studies report high levels of LS restrictions [28, 29] and restricted LS was associated with similar characteristics as in community-dwelling older adults [26, 28–30]. Still, this insubstantial evidence has limitations: LS was not the main outcome investigated [28, 29]; only residents using wheelchairs for mobility were included [30]; and none of the studies were specific to residents with dementia. Our aim was to identify LS trajectories from time of admission in NH residents with dementia and explore individual and environmental characteristics associated with LS over time.

## Methods

### Study design, setting and participants

This is a longitudinal multicentre study based on data from the Resource Use and Disease Course in dementia-Nursing Home cohort (REDIC-NH). The study procedure, methods, attrition, and eligible-but-not-included analyses have been reported in detail previously [31]. Briefly, data were collected from 47 NHs across 35 municipalities in Norway by NH health workers in collaboration with research nurses. Baseline data were collected within 1 month of admission (March 2012–November 2014); follow-up was conducted every 6 months for 36 months; and the last data were collected in May 2017. Residents were consecutively included at admission to NHs if they were 65 years or older and had an expected NH stay of more than 4 weeks. Additionally, younger persons with an established dementia diagnosis were included. Residents with life expectancy less than 6 weeks were excluded. In total, 696 residents were included at baseline in REDIC-NH. Participation was based on informed consent by the resident or next of kin if the resident was unable to provide consent. This study was approved by the Regional Ethics committee for Medical Research in South-Eastern Norway (2011/1738a).

**Fig. 1:**
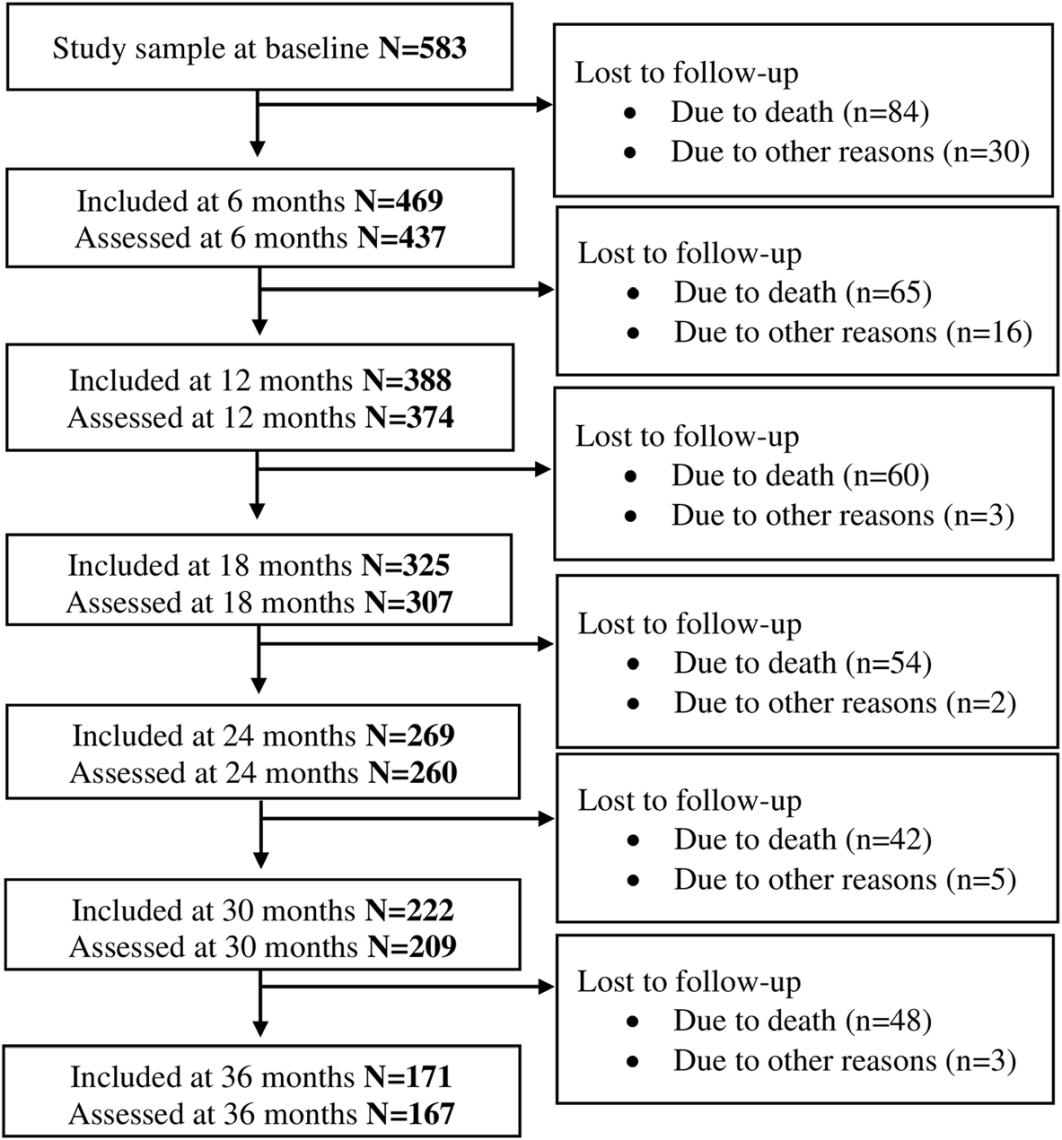
Flowchart of residents through the study

At admission, based on all data collected, two physicians (SB & GS) independently diagnosed dementia according to the International Classification of Diseases, version 10, research criteria (ICD-10) [32]. If consensus was not reached, a third physician was consulted. At admission, 583 residents were diagnosed with dementia and included in this study. A flowchart of residents through the study is presented in Fig. 1. Losses to follow-up include death and other reasons (e.g., NH withdrew, patient moved to another NH or returned home). Some residents had missing data due to incomplete follow-up assessments, and missing assessments might be due to temporary severe illness or hospitalization at the time of assessment.

### Measures

LS was assessed using the Nursing Home Life-Space Diameter (NHLSD) [5]. The NHLSD is a proxy assessment completed by NH staff based on the resident’s movement during the previous 2 weeks. The score evaluates the extent of the resident’s movement (diameter): (1) within resident’s room, (2) within unit, (3) outside unit, and (4) outside the facility; and frequency of movement: (0) never, (1) less than weekly, (2) at least weekly, (3) > 2 times/week, (4) 1–3 times/day, and (5) > 3 times/day. Composite NHLSD scores were calculated as 1(*diameter 1* × *frequency 1*) + 2(*diameter 2* × *frequency 2*) + 3(*diameter 3* × *frequency 3*) + 4(*diameter 4* × *frequency 4*), and the score ranges from 0 to 50, 0 signifying being bedbound and 50 signifying leaving the facility daily [5]. A higher NHLSD score indicates a wider LS; a lower score indicates a more restricted LS. The NHLSD demonstrates good feasibility in NH residents [33] and was found to have high intra- and inter-rater reliability and good validity [5].

Individual characteristics including age, sex, and education were assessed at admission, and general medical health, number of medications, physical performance, pain, dementia severity, and NPS were assessed at admission and follow-ups.

General medical health was assessed with the General Medical Health Rating (GMHR) [34], dichotomized as excellent/good vs fair/poor. Number of medications was collected from the NH records, based on counts of medications described with the Anatomical Therapeutic Chemical Classification System for coded medications [35]. Physical performance was assessed with the Short Physical Performance Battery (SPPB), a test comprising a balance, walking, and chair-stand test that generates a total score of 0–12 [36]. Pain was assessed with the two-part Mobilization-Observation-Behaviour-Intensity-Dementia Pain Scale (MOBID-2), musculoskeletal pain (Part 1, 0–50) and internal-organ pain (Part 2, 0–50) [37]. The Clinical Dementia Rating Sum of Boxes (CDR-SOB) (0–18), a global rating scale, was used to assess dementia severity [38, 39]. Finally, the Neuropsychiatric Inventory-Nursing Home version (NPI-NH) [40], categorized as subsyndrome scores for agitation (agitation/aggression, disinhibition, irritability, 0–36); psychosis (delusions and hallucinations, 0–24); affective symptoms (depression and anxiety, 0–24); and apathy (apathy, 0–12) based on a previous factor analysis [41] was used to assess NPS.

Environmental characteristics including staff-to-resident daytime ratio, unit size (unit defined as a group of residents living in a common area and having their own care staff during the day), and quality of the physical environment were assessed at one time point between October 2013 and December 2014. The collection of environmental characteristics in REDIC-NH has been described in detail previously [42]. Staff-to-resident daytime ratio and unit size (number of residents) were collected through questionnaires and interviews with the NH unit’s head nurse. The Special Care Unit Environmental Quality Scale (SCUEQS), comprising 18 items measuring maintenance, cleanliness, safety, lighting, physical appearance/homelikeness, orientation/cueing, and noise (0–41), was used to assess the quality of the physical environment [25].

### Statistical analysis

Individual and environmental characteristics were described as means and standard deviations (SD) or as frequencies and percentages. If one of the three items on the SPPB was missing, the total score was calculated as the sum of the two non-missing scores plus their average [43]. Missing values on the CDR-SOB, MOBID-2 Part 1 and Part 2, and the NPI-NH were imputed for cases with fewer than 50% missing items. Empirical distribution for each item was generated, and random values drawn from it were used to replace the missing value. No imputation was conducted for missing values on any other characteristics.

A growth mixture model (GMM) was estimated to identify potential groups of residents following distinct LS trajectories. Cases with missing NHLSD information at baseline were excluded from analyses. The number of groups was determined with Akaike’s Information Criterion. In addition, we aimed for reasonable group sizes, non-overlapping confidence intervals of trajectories, and average within-group probabilities of at least 0.80. Next, bivariate and multiple nominal regression analysis for hierarchical data was performed to assess whether individual and environmental characteristics measured at baseline were associated with group-belonging.

To identify characteristics associated with overall LS trend, a linear mixed model (LMM) was estimated. The model with fixed effects for non-linear time was estimated first. Next, characteristics were included one at a time, as additional fixed effects, together with the interaction between time and characteristic. Lastly, a multiple model including all characteristics and corresponding interactions was estimated and reduced for excessive interactions using Akaike’s Information Criterion. Significant interactions are illustrated in Supplementary Figure S1B.

All models contained random effects for residents nested within NH units. Only cases with no missing values on covariates were included in the regression analyses. In contrast to complete case models with respect to outcome variable, both GMM and LMM include data from all participants as well as from dropouts. Results with *p*-values below 0.05 were considered statistically significant. The statistical analyses were performed using IBM SPSS V25, SAS V9.4, and STATA V14.

## Results

At admission, the mean age of residents with dementia was 84.1 years (SD 7.5), 64.5% were female, and daytime staff-to-resident ratio was 0.32 (SD 0.10) (Table 1).

**Table 1:** Descriptive characteristics at baseline (*N* = 583)

	Statistics	N	Min–Max
**Age,** mean (SD)	84.1 (7.5)	583	50–105
**Sex,** male, n (%)	207 (35.5)	583	–
**Education (y),** mean (SD)	8.3 (2.9)	428	0–25
**GMHR Poor,** n (%)	280 (50.3)	557	–
**Medication (n),** mean (SD)	5.7 (3.1)	583	0–17
**NHLSD,** mean (SD)	25.4 (12.8)	541	0–50
**SPPB,** mean (SD)	4.3 (3.6)	531	0–12
**MOBID-2 P1,** mean (SD)	4.8 (6.5)	568	0–39
**MOBID-2 P2,** mean (SD)	3.5 (4.8)	567	0–27
**CDR-SOB,** mean (SD)	11.3 (3.6)	578	1–18
**NPI agitation,** mean (SD)	4.5 (7.3)	582	0–36
**NPI psychosis,** mean (SD)	1.9 (4.1)	581	0–24
**NPI affective,** mean (SD)	3.9 (5.9)	581	0–24
**NPI apathy,** mean (SD)	1.3 (2.7)	582	0–12
**Staff-to-resident ratio,** mean (SD)	0.32 (0.1)	583	0.15–1.0
**Unit size,** mean (SD)	10.8 (4.8)	583	3–30
**SCUEQS,** mean (SD)	25.3 (4.7)	564	13–35

*SD* Standard Deviation, *GMHR* General Medical Health Rating (dichotomized excellent/good versus fair/poor), *NHLSD* Nursing Home Life-Space Diameter, *SPPB* Short Physical Performance Battery (0–12), *MOBID-2* Mobilization-Observation-Behaviour-Intensity-Dementia Pain Scale Part 1 (0–50) and Part 2 (0–50), *CDR-SOB* Clinical Dementia Rating Sum of Boxes (0–18), *NPI* Neuropsychiatric Inventory agitation (agitation/aggression, disinhibition, irritability, 0–36), psychosis (delusions, hallucinations, 0–24), affective symptoms (depression, anxiety, 0–24) and apathy (apathy, 0–12), *SCUEQS* Special Care Unit Environmental Quality Scale (0–41)

### LS trajectories

**Fig. 2:**
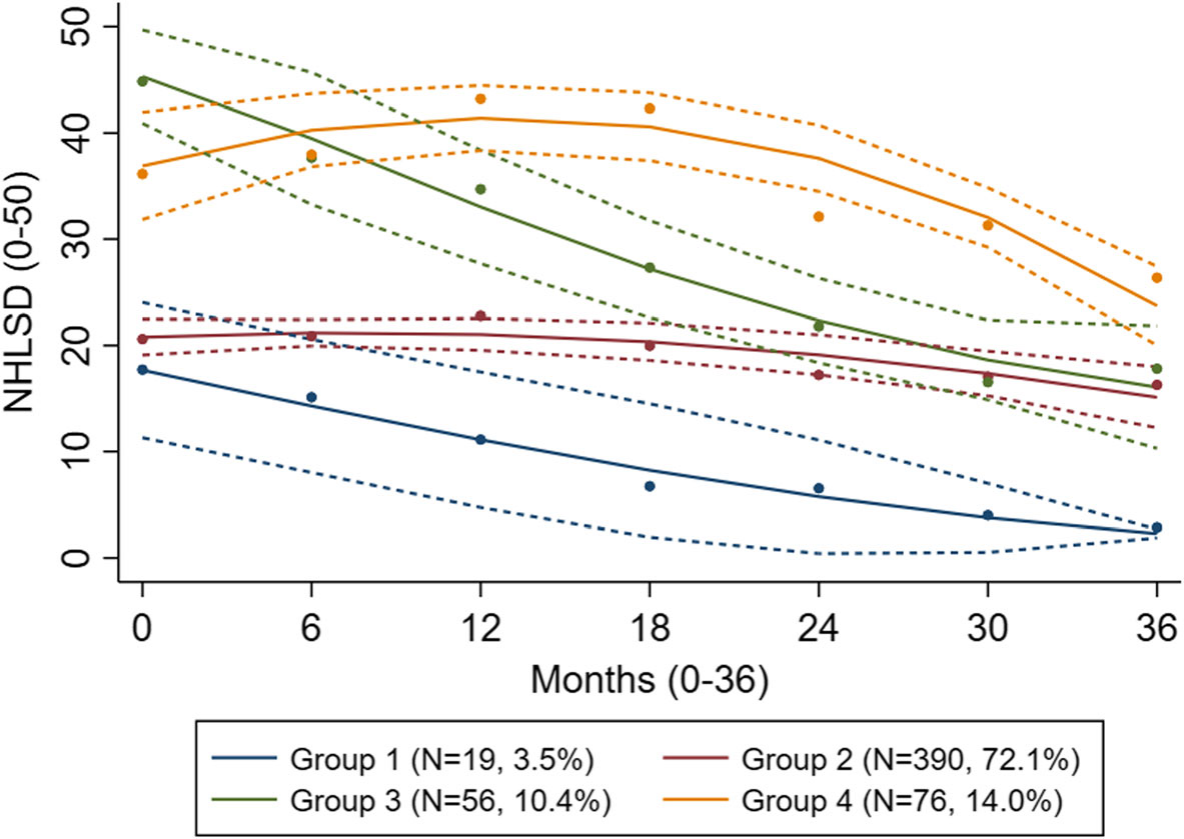
Estimated life-space trajectories (*N* = 541). NHLSD: Nursing Home Life-Space Diameter (0–50)

We identified four groups of residents with distinct LS trajectories and applied the following labels: Group 1 (*n* = 19, 3.5%); Group 2 (*n* = 390, 72.1%); Group 3 (*n* = 56, 10.4%); Group 4 (*n* = 76, 14.0%) (Fig. 2, Supplementary Table S1).

NHLSD score ranges are listed in Supplementary Table S2. At admission, the LS of Group 1 residents was restricted to within their unit (NHLSD = 17.5) and progressed to decline linearly. At the end of the study, Group 1 residents had the most restricted LS of all groups, corresponding to never moving outside their bedroom. Residents in Group 2 moved outside their unit less than weekly (NHLSD = 20.7) at admission and showed a non-linear but rather stable trajectory toward never leaving their unit by the end of the study. Group 3 residents had widest LS at admission, corresponding to moving outside the facility 1–3 times daily (NHLSD = 48.6), but their LS decreased steeply and overlapped with that of Group 2 at the end of the study. At admission, residents in Group 4 moved outside their unit more than three times daily but left the facility less than weekly (NHLSD = 37.4). Their trajectory continued to increase up to 1.5 years before declining, but maintaining the widest LS of all four groups, corresponding to moving outside their unit at least weekly.

In the multiple model older age at admission was associated with lower odds of being in Group 3 (*p* < 0.001) or Group 4 (*p* = 0.005) compared to Group 2. Additionally, better physical performance (SPPB) at admission was associated with higher odds of being in Group 3 (*p* = 0.001) or Group 4 (*p* = 0.001) compared to Group 2. Fewer medications (*p* = 0.04), less severe dementia (*p* = 0.002), and more agitation (*p* = 0.002) at baseline were associated with higher odds of being in Group 4 compared to Group 2 (Table 2).

**Table 2:** Baseline characteristics within groups (*N* = 541) and associations with group-belonging (*N* = 453^a^), Group 2 as reference

	Descriptive characteristics	Bivariate model		Multiple model	
		OR (95% CI)	***p***-value	OR (95% CI)	***p***-value
**Age,** mean (SD)					
Group 1	84.4 (9.7)	0.99 (0.92; 1.06)	0.69	0.99 (0.92; 1.07)	0.85
Group 2	85.2 (6.7)	1		1	
Group 3	79.4 (7.7)	0.90 (0.86; 0.94)	*< 0.001*	0.91 (0.87; 0.95)	***< 0.001***
Group 4	80.9 (8.8)	0.93 (0.89; 0.96)	*< 0.001*	0.94 (0.90; 0.98)	***0.005***
**Sex (male),** n (%)					
Group 1	6 (31.6)	1.21 (0.43; 3.43)	0.72	1.50 (0.49; 4.60)	0.48
Group 2	132 (33.8)	1		1	
Group 3	24 (42.9)	1.78 (0.96; 3.30)	0.07	1.45 (0.72; 2.92)	0.30
Group 4	30 (39.5)	1.21 (0.69; 2.12)	0.50	0.98 (0.51; 1.89)	0.96
**GMHR Poor,** n (%)					
Group 1	6 (33.3)	0.51 (0.18; 1.43)	0.20	0.29 (0.08; 1.05)	0.059
Group 2	204 (54.4)	1		1	
Group 3	19 (35.8)	0.48 (0.25; 0.90)	*0.02*	1.01 (0.47; 2.18)	0.99
Group 4	4 (40.5)	0.54 (0.31; 0.93)	*0.03*	1.32 (0.66; 2.64)	0.42
**Medication,** mean (SD)					
Group 1	5.6 (3.2)	0.96 (0.82; 1.14)	0.67	0.99 (0.82; 1.19)	0.90
Group 2	5.9 (3.1)	1		1	
Group 3	5.0 (3.3)	0.92 (0.83; 1.03)	0.14	0.98 (0.86; 1.11)	0.74
Group 4	4.7 (2.8)	0.86 (0.78; 0.95)	*0.003*	0.88 (0.79; 0.99)	***0.04***
**SPPB,** mean (SD)					
Group 1	3.1 (2.8)	0.96 (0.81; 1.13)	0.59	0.92 (0.76; 1.11)	0.38
Group 2	3.6 (3.2)	1		1	
Group 3	6.5 (4.1)	1.28 (1.17; 1.40)	*< 0.001*	1.19 (1.07; 1.33)	***0.001***
Group 4	6.4 (3.8)	1.28 (1.18; 1.39)	*< 0.001*	1.18 (1.07; 1.30)	***0.001***
**MOBID-2 P1,** mean (SD)					
Group 1	6.9 (7.9)	1.02 (0.95; 1.09)	0.60	1.02 (0.94; 1.11)	0.59
Group 2	5.3 (6.7)	1		1	
Group 3	2.9 (5.0)	0.90 (0.84; 0.97)	*0.006*	0.95 (0.87; 1.04)	0.29
Group 4	2.5 (4.3)	0.89 (0.83; 0.95)	*0.001*	0.93 (0.86; 1.01)	0.08
**MOBID-2 P2,** mean (SD)					
Group 1	4.2 (3.4)	1.03 (0.94; 1.13)	0.51	1.04 (0.93; 1.15)	0.51
Group 2	3.9 (5.1)	1		1	
Group 3	2.2 (3.5)	0.92 (0.84; 1.00)	0.059	0.96 (0.86; 1.06)	0.41
Group 4	2.3 (3.8)	0.93 (0.86; 1.00)	*0.047*	1.00 (0.91; 1.10)	0.99
**CDR-SOB,** mean (SD)					
Group 1	11.5 (3.8)	1.00 (0.87; 1.16)	0.97	0.98 (0.83; 1.17)	0.85
Group 2	11.6 (3.4)				
Group 3	10.8 (4.0)	0.96 (0.88; 1.05)	0.41	0.92 (0.83; 1.03)	0.15
Group 4	10.1 (3.8)	0.89 (0.82; 0.96)	*0.004*	0.85 (0.77; 0.94)	***0.002***
**NPI agitation,** mean (SD)					
Group 1	4.7 (7.6)	1.03 (0.97; 1.10)	0.38	1.00 (0.92; 1.09)	0.99
Group 2	4.3 (7.1)	1		1	
Group 3	5.1 (7.5)	1.03 (0.99; 1.07)	0.23	1.02 (0.96; 1.08)	0.57
Group 4	5.8 (8.3)	1.04 (1.00; 1.07)	*0.04*	1.06 (1.00; 1.11)	***0.04***
**NPI psychosis,** mean (SD)					
Group 1	2.8 (5.7)	1.07 (0.97; 1.17)	0.18	1.08 (0.96; 1.21)	0.22
Group 2	1.7 (4.0)	1		1	
Group 3	2.2 (4.5)	1.04 (0.98; 1.12)	0.20	1.04 (0.94; 1.14)	0.44
Group 4	2.1 (4.7)	1.05 (0.99; 1.11)	0.13	1.05 (0.97; 1.14)	0.65
**NPI affective,** mean (SD)					
Group 1	4.4 (6.1)	1.02 (0.94; 1.10)	0.67	1.02 (0.94; 1.12)	0.63
Group 2	4.1 (6.1)	1		1	
Group 3	4.2 (5.9)	1.01 (0.96; 1.06)	0.84	1.00 (0.94; 1.07)	0.93
Group 4	3.2 (5.5)	0.97 (0.93; 1.03)	0.31	0.97 (0.91; 1.03)	0.32
**NPI apathy,** mean (SD)					
Group 1	1.6 (2.5)	0.96 (0.79; 1.16)	0.64	0.92 (0.75; 1.13)	0.42
Group 2	1.6 (3.0)	1		1	
Group 3	1.0 (2.3)	0.92 (0.81; 1.05)	0.22	0.95 (0.83; 1.10)	0.50
Group 4	0.6 (2.0)	0.79 (0.67; 0.94)	*0.008*	0.86 (0.72; 1.02)	0.09
**Staff-to-resident ratio,** mean (SD)					
Group 1	0.31 (0.06)	0.78 (0.00; 390.8)	0.94	0.35 (0.00; 532.6)	0.78
Group 2	0.32 (0.08)	1		1	
Group 3	0.34 (0.13)	4.75 (0.25; 91.3)	0.30	0.34 (0.01; 11.7)	0.55
Group 4	0.32 (0.08)	1.80 (0.09; 35.2)	0.70	0.30 (0.01; 8.9)	0.49
**Unit size,** mean (SD)					
Group 1	13.1 (6.7)	1.04 (0.95; 1.13)	0.39	1.05 (0.96; 1.16)	0.29
Group 2	11.2 (4.9)	1		1	
Group 3	9.1 (3.1)	0.86 (0.77; 0.97)	*0.01*	0.91 (0.81; 1.02)	0.10
Group 4	10.0 (4.4)	0.89 (0.81; 0.97)	*0.01*	0.94 (0.86; 1.02)	0.15
**SCUEQS,** mean (SD)					
Group 1	25.2 (4.6)	0.99 (0.88; 1.10)	0.81	1.00 (0.89; 1.12)	0.97
Group 2	25.0 (4.7)	1		1	
Group 3	25.9 (4.2)	1.03 (0.97; 1.11)	0.33	0.99 (0.91; 1.06)	0.69
Group 4	26.9 (4.7)	1.10 (1.03; 1.17)	*0.003*	1.06 (0.99; 1.14)	0.12

Results of nominal regression: bivariate models include one characteristic at a time, multiple model includes all characteristics simultaneously

^a^Cases with at least one missing value on covariates were excluded (Group 1 *n* = 16, Group 2 *n* = 326, Group 3 *n* = 47, and Group 4 *n* = 64)

*OR* Odds Ratio, *CI* Confidence Interval, *SD* Standard Deviation, *GMHR* General Medical Health Rating (dichotomized excellent/good versus fair/poor), *SPPB* Short Physical Performance Battery (0–12), *MOBID-2* Mobilization-Observation-Behaviour-Intensity-Dementia Pain Scale Part 1 (0–50) and Part 2 (0–50), *CDR-SOB* Clinical Dementia Rating Sum of Boxes (0–18), *NPI* Neuropsychiatric Inventory agitation (agitation/aggression, disinhibition, irritability, 0–36), psychosis (delusions, hallucinations, 0–24), affective symptoms (depression, anxiety, 0–24), and apathy (apathy, 0–12), *SCUEQS* Special Care Unit Environmental Quality Scale (0–41)

### Characteristics associated with LS over time

In the multiple model, younger age (*p* < 0.001), having good compared to poor general medical health (*p* = 0.008), better physical performance (SPPB, *p* < 0.001), more agitation (NPI agitation) (*p* = 0.005), less apathy (NPI apathy) (*p* < 0.001), and living in a smaller unit (*p* = 0.003) were associated with on average wider LS throughout the study period (Table 3). Overall, more severe dementia (CDR-SOB) was associated with a more restricted LS, but this association varied with time (*p* = 0.004), i.e., the association became stronger from admission onward but levelled out toward the end of the follow-up period (Table 3, Supplementary Figure S1).

**Table 3:** Characteristics associated with life-space over time, *N* = 1581^a^

Characteristic	Bivariate model		Multiple model	
	Regr.coeff. (SE)	***p***-value	Regr.coeff. (SE)	***p***-value
Time	0.07 (0.07)	0.31	1.16 (0.24)	*< 0.001*
Time x Time	−0.01 (0.002)	*< 0.001*	−0.03 (0.008)	*< 0.001*
**Age**	−0.59 (0.07)	*< 0.001*	−0.31 (0.05)	***< 0.001***
Age x Time	0.02 (0.01)	*0.02*		
Age x Time x Time	−0.0002 (0.003)	0.57		
**Sex (male)**	0.45 (1.14)	0.70	−0.21 (0.80)	0.79
Sex x Time	0.15 (0.15)	0.32		
Sex x Time x Time	−0.007 (0.005)	0.15		
**GMHR Poor**	−4.60 (0.95)	*< 0.001*	−1.63 (0.61)	***0.008***
GMHR Poor x Time	0.05 (0.15)	0.73		
GMHR Poor x Time x Time	− 0.001 (0.005)	0.80		
**Medication**	−0.31 (0.16)	*0.050*	−0.003 (0.10)	0.98
Medication x Time	0.05 (0.02)	*0.04*		
Medication x Time x Time	−0.001 (0.007)	0.13		
**SPPB**	1.24 (0.13)	*< 0.001*	0.84 (0.10)	***< 0.001***
SPPB x Time	0.001 (0.02)	0.96		
SPPB x Time x Time	−0.00002 (0.00006)	0.97		
**MOBID-2 P1**	−0.32 (0.08)	*< 0.001*	−0.09 (0.05)	0.059
MOBID-2 P1 x Time	0.005 (0.01)	0.65		
MOBID-2 P1 x Time x Time	−0.00007 (0.0003)	0.82		
**MOBID-2 P2**	−0.22 (0.11)	*0.04*	−0.07 (0.07)	0.29
MOBID-2 P2 x Time	−0.002 (0.02)	0.92		
MOBID-2 P2 x Time x Time	−0.00007 (0.0005)	0.89		
**CDR-sob**	−0.26 (0.13)	0.051	−0.14 (0.13)	0.27
CDR-sob x Time	−0.09 (0.02)	*< 0.001*	−0.08 (0.02)	***< 0.001***
CDR-sob x Time x Time ^b^	0.002 (0.0006)	*0.003*	0.002 (0.0006)	***0.004*** ^**b**^
**NPI agitation**	0.16 (0.07)	*0.02*	0.12 (0.04)	***0.005***
Agitation x Time	−0.007 (0.009)	0.44		
Agitation x Time x Time	−0.0001 (0.0003)	0.70		
**NPI psychosis**	0.22 (0.11)	0.051	0.04 (0.08)	0.63
Psychosis x Time	−0.03 (0.02)	0.058		
Psychosis x Time x Time	−0.0004 (0.0005)	0.47		
**NPI affective**	0.07 (0.08)	0.43	−0.02 (0.06)	0.70
Affective x Time	−0.02 (0.01)	0.12		
Affective x Time x Time	0.0001 (0.0004)	0.71		
**NPI apathy**	−0.37 (0.17)	*0.03*	−0.39 (0.10)	***< 0.001***
Apathy x Time	−0.02 (0.03)	0.56		
Apathy x Time x Time	0.0001 (0.0008)	0.88		
**Staff-to-resident ratio**	5.11 (6.95)	0.46	−3.58 (4.37)	0.41
Staff-to-resident ratio x Time	−0.97 (0.84)	0.25		
Staff-to-resident ratio x Time x Time	0.03 (0,03)	0.33		
**Unit size**	−0.69 (0.14)	*< 0.001*	−0.30 (0.10)	***0.003***
Unit size x Time	0.02 (0.02)	0.15		
Unit size x Time x Time	0.00004 (0.00005)	0.94		
**SCUEQS**	0.44 (0.13)	*0.001*	0.15 (0.09)	0.08
SCUEQS x Time	−0.001 (0.02)	0.92		
SCUEQS x Time x Time	−0.0001 (0.0005)	0.73		

^a^Based on the number of residents assessed with Nursing Home Life-Space Diameter (NHLSD) at the 7 time points. Cases with at least one missing value on covariates were excluded (n_0_ = 453, n_6_ = 318, n_12_ = 256, n_18_ = 197, n_24_ = 153, n_30_ = 116, n_36_ = 88). ICC = 22.3%

^b^Change in association in time between life-space and CDR-SOB is illustrated in Supplementary Figure S1B

*SE* Standard Error, *GMHR* General Medical Health Rating (dichotomized excellent/good versus fair/poor), *SPPB* Short Physical Performance Battery, *MOBID-2* Mobilization-Observation-Behaviour-Intensity-Dementia Pain Scale Part 1 and Part 2, *CDR-SOB* Clinical Dementia Rating Sum of Boxes, *NPI* Neuropsychiatric Inventory agitation (agitation/aggression, disinhibition, irritability), psychosis (delusions, hallucinations), affective symptoms (depression, anxiety), and apathy (apathy), *SCUEQS* Special Care Unit Environmental Quality Scale

## Discussion

To the authors’ best knowledge, this is the first study to identify LS trajectories from time of NH admission, and explore individual and environmental characteristics associated with LS over time.

In this study, NH residents with dementia followed four distinct LS trajectories, but most experienced rather stable LS (Group 2), corresponding to moving outside the unit less than weekly at admission to never after 3 years. Thus, most NH residents’ daily lives unfolded within their unit. This finding aligns with the average LS of NH residents reported in cross-sectional studies [26, 28, 29]. Previous cross-sectional studies of LS in NH residents reported large variability in their measures, describing them as heterogeneous [26, 28, 29]. Although Group 2 was, by far, the largest, we identified three other groups, underscoring the presence of heterogeneity in our data as well.

Residents in Group 1 had the most restricted LS at admission, which continued to decline. After 3 years, this group’s LS corresponded with that of residents never moving outside their room. No characteristics measured at admission were significantly associated with belonging to Group 1 compared to belonging to Group 2. Not leaving the room could be associated with characteristics not measured in this study, such as specific diseases or conditions characterized by severe physical impairments and being bedridden. Being confined, this group stands out as especially vulnerable for inactivity. Lack of physical activity is associated with several risk factors for multiple negative health outcomes and reduced quality of life [44], underpinning the importance of facilitating LS outside the room for this group.

Of the four groups, Group 3 residents started with the widest LS but progressed to a substantial decline over time. Compared to Group 2, residents in Group 3 were younger and had better physical performance at baseline, characteristics positively associated with LS in NH residents [26, 28, 29] and community-dwelling older adults [7, 11]. LS in NHs is strongly determined by daily routines and activities [26], which should facilitate a stable wide LS in this group. These residents might have experienced rapid progression in other underlying diseases, or developed diseases limiting mobility, such as stroke or impaired balance leading to falls, that were not measured in this study. Nonetheless, the identification of residents with a high level of LS at admission appears to be an important factor for targeting individuals at risk of extensive loss of LS.

Residents in Group 4 were the only ones who demonstrated increased LS following admission and maintained the widest LS of the four groups. Group 4 residents were younger, had better physical performance and less severe dementia at admission compared to Group 2 residents. These three characteristics are associated with wider LS levels across populations [12, 13, 26, 28, 29]. These residents might have responded well to transitioning to NH care and benefited from the aid, services, and activities provided, facilitating them to increase and maintain their wide LS over time. Having more agitation was also associated with belonging to Group 4, compared to Group 2, at admission. Transition into a NH might also be experienced as distressing, driving agitation. Restless and pacing are often behavioural expressions of agitation [45] and might be an alternative explanation for the increased LS in Group 4 in the beginning of the study period. That these residents maintained a wide LS might also be explained by characteristics not assessed, such as visitors encouraging participation and venturing across NH life zones.

Younger age, having good compared to poor general medical health, less severe dementia, better physical performance, less apathy, more agitation, and living in a smaller unit were associated with on average wider LS throughout the study period. Associations with age, comorbidity, and dementia severity are supported by previous studies’ findings of community-dwelling older adults [7, 12, 14]. However, these characteristics are generally not modifiable or reversible. By contrast, physical performance and apathy, also previously reported as associated with LS [15, 26], are potentially modifiable.

Lack of initiative and motivation is an expression of apathy; thus, these symptoms are likely to negatively influence LS. Non-pharmacological interventions are recommended for treating NPS [46], and physical exercise interventions have shown positive benefits for apathy and physical performance in NH residents with dementia [47]. Physical performance was associated with group-belonging as well as LS over time, and a recent study by Jansen et al. (2018) showed that a physical exercise intervention to increase physical performance, in turn, increased LS [48]. Such interventions might facilitate more daily physical activity and prevent adverse health outcomes associated with inactivity. As mentioned previously, more agitation was associated with a wider LS group-belonging (Group 4). Additionally, more agitation was associated with wider LS over time. Agitation is more common as dementia severity increases [49], and with decreasing levels of physical activity [50]. Some authors use LS as a proxy measure of physical activity [29]. Following this line of thought, residents with a wider LS should have less agitation. Agitation is however sometimes the result of unmet needs, confusion, and physical and emotional discomfort, with restlessness and pacing as the behavioural expression [45]. If this is the case, it may explain why agitation is associated with a wider LS. For the persons’ quality of life, identification and management of agitation is essential, even if it might result in a more restricted LS.

Environmental characteristics are accessible and potentially modifiable features that influence mobility within NHs, and higher-quality physical environments have been associated with improved activities of daily life and reduction in NPS [25, 27]. However, we did not detect an association between physical environment and LS in our study. LS in NHs is strongly related to daily routines [26], and mealtimes provided in dining areas represent a key focal activity in residents’ lives [27]. Stable daily routines and mealtimes provided within the unit, could explain why most residents maintained a stable LS and why daily life unfolded within their unit (Group 2). A perceived lack of environmental barriers can facilitate LS [14]. In staff-involved or supervised daily routines, the physical environment might not be perceived as a barrier and therefore not associated with LS in our study. Instead, we showed that living in a smaller unit was associated with a wider LS over time. Such an environment might be more conducive to social bonding and familiarity with peers and proximity to staff, and physical distances between life zones might be shorter, encouraging the utilisation of LS. Additionally, environmental characteristics which we did not assess such as type of activities offered and where, unsuitably designed outdoor areas, and physical barriers (e.g., locked doors, stairs) could affect LS and explain why most residents’ LS is within the unit (Group 2). Greater variability and flexibility in types of activities provided, offered in accessible areas outside the unit could facilitate residents’ opportunities for expanding their LS. Finally, social network and support is a driver of LS [3, 8–10], and in the NH setting visitors encouraging participation and venturing across NH life zones, might affect the LS of a resident. Unfortunately, we did not collect this type of information. LS is a multi-layered construct and despite the broad assessment battery applied, several characteristics that could have have affected LS were not measured. This is a limitation of our study. Some further methodological issues should also be considered.

Firstly, mortality in this population is high and the major reason for loss to follow-up (Fig. 1). The statistical approaches taken, in contrast to complete case methods, use all available data at all time points, and both LMM and GMM take dropouts into account and are not limited to survivors. Still, as the number of residents decreases during the study period, the uncertainty of the estimates increases. Secondly, only cases with no missing values on covariates were included in the LMM and nominal regression analyses. Due to a high number of missing values, “years of education” was not included in these analyses as a covariate. When included, the sample size was substantially reduced; however, overall LS trend, number of distinct groups, and shape of trajectories remained. Further, new technology has introduced sensor-based assessments of LS, which might be more sensitive and specific than proxy rated LS through questionnaires. Additionally, the main outcome measure, NHLSD, lacks established cut-off values for meaningful change, and the Norwegian translation of the instrument has not been tested for reliability. However, we believe that the standardized and comprehensive training of all project nurses and health workers [31] ensured limited variability between assessors. This study has several strengths, including its comprehensive assessment battery, assessments performed on a regular basis (every 6 months), and wide geographic recruitment area. A major advantage of the NHLSD is its ease of administration. Finally, this study has several novelties, including being the first to identify LS trajectories and explore associated characteristics over time, in an observational longitudinally design in the NH setting.

## Conclusion

From NH admission most NH residents’ LS trajectory remained stable, and their daily lives unfolded within their unit. Better physical performance and less apathy emerged as potentially modifiable characteristics associated with wider LS over time. Future studies are encouraged to determine whether LS trajectories in NH residents are modifiable, and we suggest that future research further explore the impact of environmental characteristics beyond the physical environment.

## Supplementary Information

**Additional file 1: Figure S1.** Unadjusted overall trend in life-space (A) and change in association in time between life-space and CDR-SOB (B). NHLSD: Nursing Home Life-Space Diameter (0–50); CDR-SOB: Clinical Dementia Rating Scale Sum of Boxes. **Table S1.** Estimated life-space trajectories (*N* = 541). **Table S2.** Nursing Home Life-Space Diameter (NHLSD) score ranges.

## Data Availability

The data that support the findings of this study are available for researchers in cooperation with the data owner, Research Centre for Age-Related Functional Decline and Disease, Innlandet Hospital Trust. Further information can be found at. https://sykehuset-innlandet.no/avdelinger/alderspsykiatrisk-avdeling/forskningssenteret-for-aldersrelatert-funksjonssvikt-og-sykdom

## Abbreviations

LS: Life-space
NH: Nursing home
NPS: Neuropsychiatric symptoms
REDIC-NH: Resource use and disease course in dementia-Nursing Home
NHLSD: Nursing Home Life-Space Diameter
GMHR: General Medical Health Rating
SPPB: Short Physical Performance Battery
MOBID: Mobilization-Observation-Behaviour-Intensity-Dementia pain scale
CDR-SOB: Clinical Dementia Rating Sum of Boxes
NPI-NH: Neuropsychiatric Inventory-Nursing Home version
SCUEQS: Special Care Unit Environmental Quality Scale
GMM: Growth mixture model
LMM: Linear mixed model
SD: Standard deviation
