# Supplementary material for: Exploring life-space in the nursing home. An observational longitudinal study
Source: BMC Geriatr. 2021 Jun 29;21:396. doi: 10.1186/s12877-021-02345-0 (PMC8243900; doi:10.1186/s12877-021-02345-0)
Supplement: Supplementary file 1 — Additional file 1: Figure S1. Unadjusted overall trend in life-space (A) and change in association in time between life-space and CDR-SOB (B). NHLSD: Nursing Home Life-Space Diameter (0–50); CDR-SOB: Clinical Dementia Rating Scale Sum of Boxes. Table S1. Estimated life-space trajectories (N = 541). Table S2. Nursing Home Life-Space Diameter (NHLSD) score ranges. [file 12877_2021_2345_MOESM1_ESM.docx]

**SUPPLEMENTARY MATERIAL**

| **Supplementary Table S1** Estimated life-space trajectories (N=541) | | | | | | | | |
| --- | --- | --- | --- | --- | --- | --- | --- | --- |
| Parameter | **Group 1**  (N=19, 3.5%) | | **Group 2**  (N=390, 72.1%) | | **Group 3**  (N=56, 10.4%) | | **Group 4**  (N=76, 14.0%) | |
|  | **Regr.coeff. (SE)** | **p-value** | **Regr.coeff. (SE)** | **p-value** | **Regr.coeff. (SE)** | **p-value** | **Regr.coeff. (SE)** | **p-value** |
| Intercept | 17.52 (2.56) | <0.001 | 20.72 (0.72) | <0.001 | 48.59 (3.35) | <0.001 | 37.38 (1.90) | <0.001 |
| Linear | -0.60 (0.12) | <0.001 | 0.11 (0.10) | 0.27 | -1.47 (0.33) | <0.001 | 0.86 (0.21) | <0.001 |
| Quadratic |  |  | -0.008 (0.003) | 0.005 | 0.02 (0.008) | 0.06 | 0.03 (0.005) | <0.001 |
| Within-group probability | 0.8 | | 0.9 | | 0.8 | | 0.8 | |

| **Supplementary Table S2** Nursing Home Life-Space Diameter (NHLSD) score ranges | | | | | | | |
| --- | --- | --- | --- | --- | --- | --- | --- |
|  | **FREQUENCY** | | | | | | |
| **D**  **I**  **A**  **M**  **E**  **T**  **E**  **R** |  | **Never** | **Less than weekly** | **At least weekly** | **>2 times a week** | **1 – 3 times a day** | **>3 times a day** |
|  | **Within the resident’s room (1)** | 0 | 1–1.99 | 2–2.99 | 3–3.99 | 4–4.99 | 5 |
|  | **Within the unit (2)** | 5–6.99 | 7–8.99 | 9–10.99 | 11–12.99 | 13–14.99 | 15 |
|  | **Outside the unit (3)** | 15–17.99 | 18–20.99 | 21–23.99 | 24–26.99 | 27–29.99 | 30 |
|  | **Outside the facility (4)** | 30–33.99 | 34–37.99 | 38–41.99 | 42–45.99 | 46–49.99 | 50 |
| Composite score=1(*diameter 1 × frequency 1*) + 2(*diameter 2 × frequency 2*) + 3(*diameter 3 ×* *frequency 3*) + 4(*diameter 4 × frequency 4*) | | | | | | | |
